# Structural Aspects of Phenylalanylation and Quality Control in Three Major Forms of Phenylalanyl-tRNA Synthetase

**DOI:** 10.4061/2010/983503

**Published:** 2010-06-27

**Authors:** Liron Klipcan, Igal Finarov, Nina Moor, Mark G. Safro

**Affiliations:** ^1^Department of Structural Biology, Weizmann Institute of Science, P.O. Box 26, 76100 Rehovot, Israel; ^2^Institute of Chemical Biology and Fundamental Medicine, Siberian Branch of Russian Academy of Sciences (RAS), Novosibirsk 630090, Russia

## Abstract

Aminoacyl-tRNA synthetases (aaRSs) are a canonical set of enzymes that specifically attach corresponding amino acids to their cognate transfer RNAs in the cytoplasm, mitochondria, and nucleus. The aaRSs display great differences in primary sequence, subunit size, and quaternary structure. Existence of three types of phenylalanyl-tRNA synthetase (PheRS)—bacterial (*αβ*)_2_, eukaryotic/archaeal cytosolic (*αβ*)_2_, and mitochondrial *α*—is a prominent example of structural diversity within the aaRSs family. Although archaeal/eukaryotic and bacterial PheRSs share common topology of the core domains and the B3/B4 interface, where editing activity of heterotetrameric PheRSs is localized, the detailed investigation of the three-dimensional structures from three kingdoms revealed significant variations in the local design of their synthetic and editing sites. Moreover, as might be expected from structural data eubacterial, *Thermus thermophilus* and human cytoplasmic PheRSs acquire different patterns of tRNA^Phe^ anticodon recognition.

## 1. Introduction

Aminoacyl-tRNA synthetases are primary actors at the first stage of protein translation, catalyzing the attachment of the correct amino acid to its cognate tRNA in a two-step reaction [1, 2]. At the first step, amino acid is activated by ATP resulting in formation of an enzyme-bound aminoacyl-adenylate. In the second step, the amino acid moiety is transferred onto the 3′-terminal ribose of the cognate tRNA, leading to synthesis of aminoacyl-tRNA. AaRSs vary greatly in amino acid sequences, three-dimensional structures, and subunit organizations. After the three-dimensional structures of four different aaRSs from various sources were determined, analysis of the structures coupled with multiple sequence alignments led to subdivision of the aaRSs family into two different classes (Table 1) [3].It was shown that the active site of class I aaRSs is associated with a classical dinucleotide-binding Rossmann fold, while the active site of class II is formed by an antiparallel *β*-sheet flanked by helices on both sides. Another discrepancy between the classes is related to the site of amino acid attachment: class I enzymes attach the amino acid substrate to the 2′-OH group of terminal ribose, whereas class II enzymes attach the amino acid to the 3′-OH group (with PheRS being the only exception from this rule).

At the amino acid binding and recognition step, some aaRSs prior to activation face the challenge of discrimination between amino acids with closely similar chemical structures. To ensure a high level of total accuracy of protein biosynthesis, aaRSs developed an additional editing activity associated with the specific site where misacylated tRNA is hydrolyzed [4]. Considerable progress has been made in revealing the structural basis and mechanisms of noncognate amino acids discrimination. The proofreading *mechanism* of the aaRSs seems to insure *discrimination* between the correct substrate and other *amino acids.* Here we discuss peculiarity of the aminoacylation reaction in Phenylalanine-specific system (Phe-system) and implications of the phenylalanyl-tRNA synthetase (PheRS) to incorporate noncanonical amino acids into proteins.

## 2. PheRS: Structural Organization and Evolution

**Figure 1:**
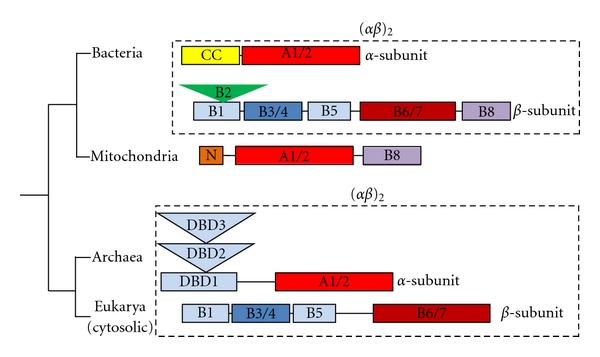
Three major forms of PheRS according to phylogenetic and structural studies: (*αβ*)_2_-heterotetrameric eubacterial; (*αβ*)_2_-heterotetrameric archaeal/eukaryotic cytoplasmic; monomeric mitochondrial. Schematic representation of *α*- and *β*-subunits in terms of structural domains.

PheRS is known to be among the most intriguing enzymes of the aaRSs family. Phylogenetic and structural studies revealed three major forms of PheRS: (a) (*αβ*)_2_-heterotetrameric eubacterial; (b) (*αβ*)_2_-heterotetrameric archaeal/eukaryotic cytoplasmic; and (c) monomeric mitochondrial (Figure 1). The heterotetrameric subunit organization of prokaryotic and eukaryotic cytoplasmic PheRSs is markedly conserved in all known species. The bacterial PheRS from *Thermus thermophilus* HB8 with 350 residues per *α*- and 785 residues per *β*-subunit is among the well-documented class II aaRSs [5–9]. Two subunits of the *αβ*-heterodimer have no detectable sequence homology. Neither the *α*- or *β*-monomers nor the *α*_2_- or *β*_2_-dimers manifest catalytic activity of tRNA aminoacylation [10]. The *α*-subunit of PheRS contains common to class II aaRSs catalytic module (CAM, composed of domains A1 and A2), which together with the N-terminal coiled-coil fragment (CC) is involved in tRNA^Phe^ binding and aminoacylation (Figure 1). The *β*-subunit is a “large” collection of structural domains including the “catalytic-like” module (CLM, composed of domains B6 and B7) structurally similar to the CAM, but catalytically not active, two helix-turn-helix (HTH) “DNA binding-like” domains, B1 and B5. In addition, the *β*-subunit contains an “EMAP II-like” domain (B2) (similar to the anticodon-binding domain of AspRS and LysRS) and a “SH3-like” domain (B3/B4), associated with signal transduction in a number of eukaryotic proteins and with an editing activity of PheRS [7]. An anticodon binding domain (B8) with a classical RNA recognition motif that directly interacts with the anticodon loop of tRNA^Phe^ is located at the C-terminus of the *β*-subunit (Figure 1). Thus, the major role of the *β*-subunit is in recognition and binding of tRNA^Phe^. Structural analysis of the *tt*PheRS complexed with tRNA^Phe^ further demonstrated that one tRNA^Phe^ molecule interacts with all four subunits of the enzyme, thereby explaining why the enzyme is a functional (*αβ*)_2_-heterodimer [8].

A tetrameric organization is not a prerequisite for aminoacylation activity, as the monomeric mitochondrial PheRS (*hm*PheRS) is fully active [11]. *Hm*PheRS, the smallest known nuclear encoded synthetase, exhibits significant homology to bacterial PheRSs. In fact, *hm*PheRS is a chimera of the CAM of the *α*-subunit and the B8-domain from the *β*-subunit of bacterial PheRS (Figure 1). As would be expected, the 3D structure of mitochondrial enzyme revealed substantial similarity to the bacterial relative, both in the architecture of individual domains CAM and B8 and in the mode of substrate recognition [12]. However, when the catalytic core of the mitochondrial enzyme is superimposed onto CAM of *tt*PheRS-tRNA^Phe^ complex, the anticodon binding domain of* hm*PheRS interferes with the acceptor stem of tRNA^Phe^ [12]. Thus, it was hypothesized that formation of the binary *hm*PheRS-tRNA^Phe^ complex may be accompanied by considerable rearrangement of the anticodon-binding domain; and indeed, very recent biochemical and SAXS experiments corroborate this hypothesis [13]. 

Although archaeal/eukaryotic and bacterial PheRSs share common architecture of the core domains (two CAMs from the *α*-subunits and two CLMs from the *β*-subunits) implicated in formation of a four-helix bundle intersubunit interface, elongation or shortening at the N- or C-terminal extremities of the *α*- and *β*-subunits have also been detected in archaeal/eukaryotic PheRSs (Figure 1) [14, 15]. The extension at the N-terminus of the *α*-subunit in archaeal/eukaryotic PheRSs consists of three structural domains with prototypical DNA-binding folds (DNA-binding domains, DBD) [16, 17]. Two of them (DBD-1 and DBD-3) belong to a superfamily of “winged helix” DNA-binding domains (SCOP a.4.5). Furthermore, the anticodon-binding domain B8 of bacterial PheRSs is missing from archaeal/eukaryotic enzymes. This results in essential changes of the architecture of archaeal/eukaryotic enzymes and in variations of the tRNA^Phe^ binding and recognition modes as compared to bacterial PheRSs [17].

It is believed that modular design of aaRSs is a result of a patchwork assembly of different functional modules during the evolution [16]. And PheRS is probably the *“tour de force”* collection of different RNA- and DNA-binding modules assembled around the class II catalytic core. The crucial branch point on the phylogenetic tree of PheRSs is the subdivision between Bacteria and Archaea lineages. Although catalytic cores of archaeal/eukaryotic and bacterial PheRSs display similarity in their heterotetrameric organization, their anticodon binding domains, to all appearance, developed independently within the two branches. The separation into two different subclasses of PheRSs is almost universal and took place at the early stage of separation between Bacteria and Archaea. From phylogenetic analysis it follows that *hm*PheRS is the newest branch evolved from a bacterial ancestor [18]. In many aspects *hm*PheRS is unique among other class II aaRSs. First, it is the smallest known aaRS, created by massive loss of domains involved in binding of tRNA and probably of dsRNA/dsDNA, and in protein quality control. Second, it is the only known example of monomeric aaRSs containing the class II CAM. Third, significant conformational mobility of the CAM and of the anticodon-binding domain in *hm*PheRS is essential factor of the phenylalanylation activity [13]. Interestingly, the replacement of the heterotetrameric bacterial form of PheRS by the monomeric one in mitochondria appears to be universal among eukaryotes.

## 3. Selection of the Amino Acids by PheRSs: Recognition and Proofreading

**Figure 2:**
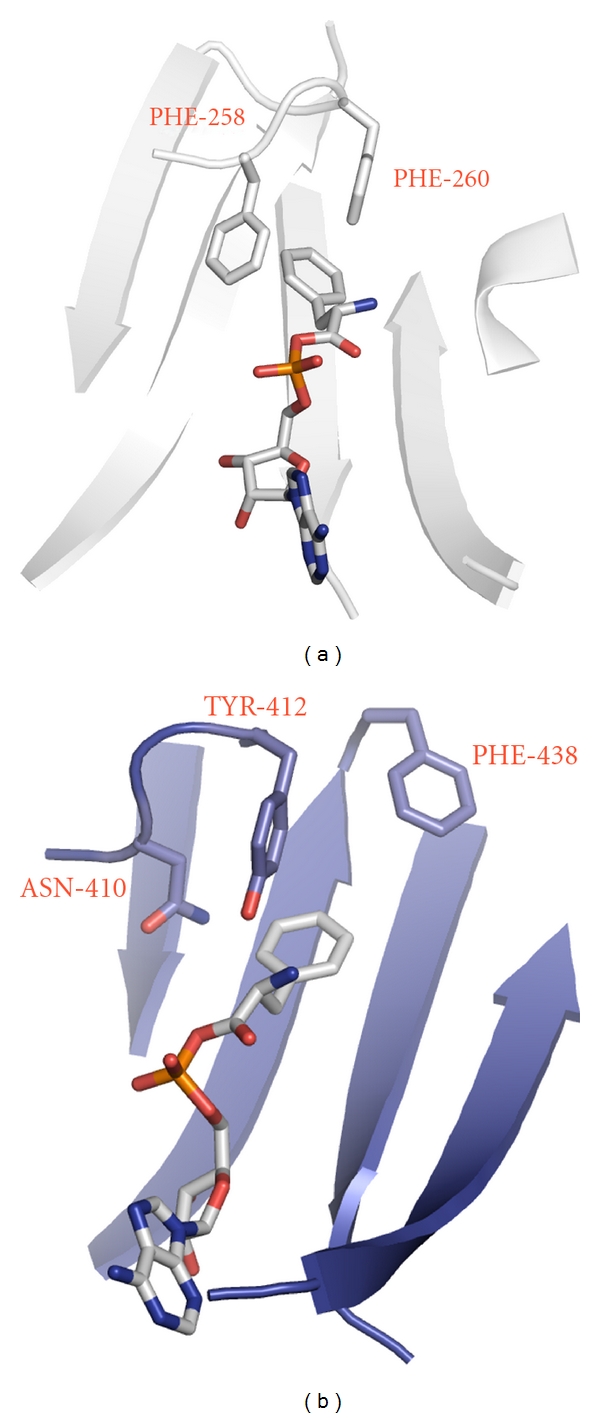
Crystal structures of *tt*PheRS and *hc*PheRS depicted in similar orientations. (a) The crystal structure of *tt*PheRS in the synthetic active site area complexed with bound phenylalanyl-adenylate. The principal protein residues forming “edge-to-face” interactions in aromatic triad are indicated. (b) The crystal structure of *hc*PheRS in the synthetic active site area. Modeling of complex with phenylalanyl-adenylate.

A distinctive feature of the bacterial PheRSs active site topology is the presence of a deep phenylalanine-binding pocket [6]. The bottom surface of the pocket is parallel to the phenyl ring of the substrate and is covered by the invariant glycines, thus providing the space required for the Phe and ATP molecules. One of the walls and the top surface of the pocket are covered by hydrophobic residues. Another wall of the pocket is built up entirely of residues, which may participate in electrostatic interactions and in hydrogen bonding. Such anisotropy in the distribution of hydrophobic and hydrophilic residues within the pocket unambiguously orients the amino and carbonyl groups of the amino acid and of the aminoacyl-adenylate (Phe-AMP) [6]. In bacterial PheRS the specific recognition of phenylalanine is achieved by hydrophobic interactions of the substrate phenyl ring and two neighboring phenyl rings of the protein (Phe*α*258 and Phe*α*260 in *tt*PheRS) making a “network” of interactions in which each aromatic pair is arranged in “edge-to-face” manner (Figure 2(a)). A given anisotropy and “network” of interactions between hydrophobic residues within the phenylalanine-binding pocket is not retained in archaeal/eukaryotic cytoplasmic PheRSs, since Asn*α*410 (in human PheRS,* hc*PheRS) substitutes for Phe*α*258 (in *tt*PheRS) and Tyr*α*412 which is invariant in all eukaryotic PheRSs substitutes for *tt*Phe*α*260 (Figure 2(b)). While the last substitution may be defined as a conservative change, then Phe*α*438 seen in *hc*PheRS instead of Val*α*286 in *tt*PheRS is not obvious at all. However, as is seen from the structure of *hc*PheRS complexed with phenylalanine, specific recognition of the small substrate still proceeds via two aromatic residues, Tyr*α*412 and Phe*α*438. Thus, the aromatic triad with “edge-to-face” type of interactions exists in *hc*PheRS as well. However, the recognition elements of *hc*PheRS are shifted from the left side of the phenylalanine-binding pocket to the right one, when compared to *tt*PheRS (Figures 2(a) and 2(b)).

Among natural amino acids only aromatic amino acids might be considered as efficient substrates for binding to PheRS, since the recognition process is essentially driven by the “network” of aromatic-aromatic interactions. The kinetic study revealed hydrophobic aromatic L-tryptophan and slightly polar aromatic L-tyrosine to compete with L-phenylalanine for the binding to PheRSs, while charged aromatic L-histidine and hydrophobic aliphatic L-isoleucine showed no inhibition effect even at 20 mM concentration [5]. Tyr is the only natural canonical amino acid misactivated by PheRSs; the catalytic efficiency of the reaction is three orders of magnitude lower as compared to that of the cognate substrate (primarily due to low affinity). The catalytic site, in this instance, is suggested to act as a “coarse sieve", discriminating noncognate amino acids at the binding and activation steps by chemical and steric factors. Final rejection of Tyr is achieved in a separate editing domain of bacterial and cytoplasmic archaeal/eukaryotic PheRSs by selective binding and hydrolysis of Tyr-tRNA^Phe^ [5, 19–21] (as described below). *Hm*PheRSs lacking such activity can stably attach Tyr to tRNA^Phe^ [22, 23]. 

*Hm*PheRS largely resembles the bacterial enzyme in architecture of the amino acid binding pocket [6, 12]. The noncognate Tyr is bound by human *hm*PheRS with a 1.7-fold higher affinity than by the bacterial PheRS [6, 23]. The structural basis of this difference might be associated with a point mutation when hydrophobic valine in the bacterial enzyme (Val*α*261 in *tt*PheRS) is substituted for polar threonine (Thr235 in *hm*PheRS). This substitution may reduce hydrophobic contacts of the Phe-substrate within the amino acid binding pocket, resulting in its lower affinity to *hm*PheRS than to *tt*PheRS (as evident from the respective *K*_*m*_ values [23]). Appearance of hydroxyl-containing Thr235 at the bottom of the *hm*PheRS amino acid binding pocket lends additional credence to the notion of PheRS active site plasticity. 

**Figure 3:**
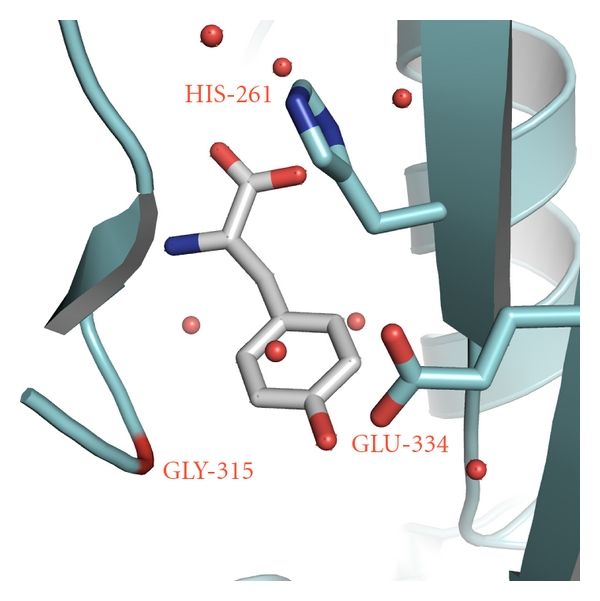
*Tt*PheRS editing site with bound Tyr [5]. The protein residues participating in direct and water-mediated (red spheres) contacts are shown.

Editing module of PheRS during evolutionary variations was specifically designed for binding and subsequent hydrolysis of Tyr-tRNA^Phe^. The early fast kinetic study of Lin et al. demonstrated that tyrosine is indeed transferred to tRNA^Phe^, and the misaminoacylated tRNA is very rapidly hydrolyzed [21]. Recently it was reported that editing activity of the bacterial and archaeal/eukaryotic PheRSs is associated with the active site located at the interface region between B3 and B4 domains in the *β*-subunit [5, 19, 20]. In *tt*PheRS the specific recognition of the OH group in *para* position of Tyr is achieved by its interactions with the O*ε*1 of Glu*β*334 and the main chain amide of Gly*β*315 (Figure 3). In accordance with the structure-based modeling Glu*β*334 and His*β*261 are considered as residues playing critical role in anchoring Tyr-tRNA^Phe^ within the editing site and its subsequent hydrolysis. It is of interest that His*β*261 together with Glu*β*323 coordinates a ‘‘catalytic” water molecule which was observed in several crystal structures of *tt*PheRS and as such may be involved in hydrolysis [5]. The presence of His*β*261, Glu*β*334, Asn*β*250, Thr*β*249, and Glu*β*323 in the vicinity of the ester bond subjected to hydrolysis is reminiscent of the active site of peptidyl-tRNA hydrolase (PTH) performing the hydrolysis of the ester bond between tRNA and the peptide [24, 25]. Residues Asn10, His20, and Asp93 considered as being crucial for PTH activity [24, 25] are similar to the PheRS triad, Asn*β*250, His*β*261, and Glu*β*323. These triads can be superimposed with r.m.s.d. of 1.4 Å for the C_*α*_ atoms in spite the fact that the proteins are not homologues. Nevertheless the clear structural similarity between two triads, biochemical experiments suggest that in PTH the triad seems to be directly involved in catalysis, while the triad in the editing site of PheRS plays more positional role rather than directly involved in catalysis. It is remarkable that both the bacterial and archaeal/eukaryotic forms of PheRS share common editing domain structures, however, PTH triad is not present in the archaeal enzyme [20]. His*β*261 appears to be replaced by Gly in archaeal/eukaryotic PheRS. At first glance, this substitution would seem to imply that the hydrolytic mechanism is different in eukaryotic cytoplasmic (as well as in archaeal) PheRSs. However, detailed inspection of the editing site shows that in place of the side chain of His*β*261 (*tt*PheRS), a given local area in *hc*PheRS is occupied by side chain of Asn*β*238 (*hc*PheRS) or Asn217 (*ph*PheRS), also capable of interacting with the phosphoester bond of the Tyr-tRNA^Phe^.

## 4. Incorporation of Non-Coding Amino Acids

The aaRSs in general demonstrate a remarkable specificity towards their cognate amino acid substrates. As a consequence, binding of odd shaped nonnatural amino acids in amino acid binding pocket have to be accompanied by substitution of several residues, respectively, [26]. Notably, despite the high level of the substrate stereospecificity, the active site of PheRS demonstrates a natural plasticity, enabling binding, activation and specific aminoacylation of some Phe surrogates. 

Exposure of the phenylalanine to reactive oxygen species (ROSs) produces multiple isomers of tyrosine—*meta*-tyrosine (*m*-Tyr), *ortho*-tyrosine (*o*-Tyr) and standard *para*-tyrosine. The first two are widely used as an index of oxidative damage in tissue proteins [27, 28]. Recently it was shown that *hm*PheRS catalyzes direct attachment of naturally occurred derivatives of phenylalanine to tRNA^Phe^ in eukaryotic cells [23]. Analysis of kinetic parameters of tRNA^Phe^ aminoacylation shows that the affinity of *hm*PheRS to *m*-Tyr is only five-fold lower than to the cognate amino acid phenylalanine. At the same time, the catalytic efficiency of tRNA^Phe^ aminoacylation with physiological Tyr is 1000-fold lower than that of the phenylalanylation reaction, primarily because of a high *K*_*m*_ value [23]. Crystal structure of *hm*PheRS complexed with *m-*Tyr revealed the highly specific network of interactions of the molecule. A fragment of unbiased electron density at the amino acid binding pocket may be unambiguously attributed to the *m*-Tyr molecule. Just as Phe and Tyr, the *m*-Tyr is involved in aromatic-aromatic interactions (see above) with two phenylalanines within the amino acid binding pocket. As compared to the Phe-substrate, *m*-Tyr is additionally stabilized by the hydrogen bonding of its OH-group in *meta*-position with the N*ε*2 atom of Gln124 (2.7 Å), and with the O*ε*2 atom of Glu159 (2.6 Å). 

As compared to bacterial and mitochondrial PheRSs, *hc*PheRS binds *m*-Tyr less efficiently (with one order of magnitude lower affinity) [23]. Modeling experiments indicate that amino acid residues of *hc*PheRS involved both in phenylalanine and *m*-Tyr binding and recognition form a local environment, which is different from those observed in *hm*PheRS and *tt*PheRS (Finarov et al., unpublished results). The distinguishable features in the amino acids environment may lead to the observed difference in the *m*-Tyr affinity. 

The substantial plasticity of the active site and the structural diversity of PheRSs from different kingdoms made these enzymes of superior tool for introduction of new amino acids into the protein polypeptide chains. Indeed, the PheRS enzyme was among the first aaRSs used to introduce Phe analogs into proteins. Ibba et al. [29] have demonstrated an attachment of the *para*-halogenated Phe analogs to tRNA and their *in vivo* incorporation into cellular proteins by the *E. coli* Ala294Gly mutant PheRS (Ala314 in *tt*PheRS) that exhibits relaxed substrate specificity. Further studies revealed that phenylalanine analogs substituted with various chemical groups (bromo-, iodo-, ethynyl-, cyano, and azido-) at *para* position were efficiently incorporated into recombinant proteins by the mutant PheRS [30, 31]. However, this mutant failed to sufficiently incorporate *p*-acetylphenylalanine into the proteins. The analysis of PheRSs 3D sructures identified another mutation that may be crucial for plasticity and active site specificity, the Thr251Gly (Val261 in *tt*PheRS). Indeed doubly mutated PheRS (T251G, A294G) as predicted by the design algorithm efficiently incorporates *in vivo p*-acetylphenylalanine into recombinant proteins [32]. The net result of these active-site mutations is formation of the space in the phenylalanine binding pocket needed to accommodate sterically demanding *para*-substituted analogs. Interestingly, the relaxed specificity of these mutants appeared to be directed mainly to the *para*-position of the amino acid substrates. However, natural plasticity of the amino acid binding pocket in* tt*PheRS allows *p*-Cl-Phe to be bound and recognized by the wild-type enzyme [5]. This suggests that engineered specificity for one enzyme may be considered as a natural one for another representative of the family. 

It was shown that mutations in the editing module may be considered as a powerful tool for the incorporation of novel amino acids. On the other side, mutations within the editing site may lead to incorporation of non-natural amino acids into the polypeptide chains and as a consequence to protein misfolding. This has been demonstrated with AlaRS: a missence mutation in the editing domain of the alanyl-tRNA synthetase gene that compromises the proofreading activity of the enzyme causes cerebellar Purkinje cell loss and ataxia [33]. Certain PheRSs are able to stably attach *para*-substituted Phe analogs to tRNA^Phe^. Considering the availability of editing activity in PheRS, appearance of misacylated tRNA^Phe^ implies that the unnatural amino acid attached to tRNA is not recognized by the hydrolytic site. It was proposed that during evolution the editing activity in PheRS is evolved against Tyr only, yet some recent observations suggest that editing site also demonstrates some degree of natural plasticity. The fact that bacterial PheRS activates *m*-Tyr, but does not stably attach the ROS-damaged amino acid to tRNA^Phe^ suggests availability of the hydrolytic activity. Contrary to bacterial PheRSs, *hc*PheRS is unable to hydrolyse* m*-Tyr-tRNA^Phe^ though it is fully active against Tyr-tRNA^Phe^. The low deacylation activity of *hc*PheRS against *m*-Tyr-tRNA^Phe^ suggests that this substrate is poorly recognized by the editing pocket of archaeal/eukaryal PheRS. Thus, editing of several different substrates by PheRS suggests that plasticity could be attributed not only to the synthetic active site, but also to the editing site, capable of binding ligands other than tyrosine. As it was reported in [34], the editing module of PheRS is capable to enhance incorporation of tyrosine derivatives into proteins when attached to tyrosyl-tRNA synthetase (TyrRS). TyrRSs from various sources have been engineered and used for the incorporation of unnatural amino acids into proteins using bacterial and eukaryotic hosts. However, these variants of TyrRS still produce Tyr-tRNA^Tyr^. Thus, the editing module of archaeal PheRS was transplanted into engineered 3-iodo-tyrosyl-tRNA synthetase (iodo-TyrRS) to edit Tyr-tRNA^Tyr^ and thereby to improve the overall specificity for 3-iodo-L-tyrosine. The engineered iodo-TyrRS-B3/B4 chimera exclusively incorporated 3-iodo-ryrosine into the specified site of a protein in the wheat germ translation cell-free system [34]. It is not clear if isolated editing module of PheRS would have adverse effect on translation *in vivo *as it edits not specifically both Tyr-tRNA^Phe^ and Tyr-tRNA^Tyr^. The most promising attack on this problem appears to be the engineering of editing domain capable to recognize specifically tRNA molecules of interest.

## 5. Conclusion Remarks

The aaRSs are a notoriously diverse family of enzymes arose early in evolution, being essential for stepwise evolution of the genetic code. Much evidence suggests that aromatic amino acids (tyrosine, tryptophan and phenylalanine) were among the last amino acids to be added to genetic code. Indeed, it seems that both PheRSs and TyrRSs continue to evolve independent tRNA identity elements since separation between bacterial and archaeal lineages. Thus, archaeal/eukaryotic TyrRS-tRNA^Tyr^ and PheRS-tRNA^Phe^ pairs do not cross-react with their bacterial counterparts. Such orthogonality can be used for the incorporation of unnatural amino acids into proteins upon introduction of engineered archaeal/eukaryotic PheRSs or TyrRSs into bacterial cells. 

At one time we were under impression that knowledge of Phe-system was adequate, and future studies of the enzyme will be referred as an applied research. However, results highlighting the structural and functional diversity of PheRSs affirm the various pathways important for biomedical research. The PheRS shows a remarkable specificity to their cognate amino acid substrate. As a result, the incorporation of non-natural amino acids often necessitates site-directed mutagenesis of the several residues within the active site area [35]. However, despite the high level of the substrate stereo-specificity in the Phe-system, structures of bacterial and mitochondrial PheRSs demonstrate a natural plasticity at the active site [5, 23], thus enabling binding and specific aminoacylation of surrogates.

Being central components of translation machinery it is plausible to propose that expression of some aaRSs show abnormal up- or downregulation in cancer. Indeed, it was shown that the level of mRNA encoded for alpha-subunit of the human PheRS is overexpressed in the lung solid tumors and acute phase chronic myeloid leukemia [36]. An enrichment-based pathway mapping of the androgen-regulated proteomic data sets revealed a significant disregulation of aminoacyl-tRNA synthetases and, *α*-subunit of PheRS in particular, indicating an increase in protein biosynthesis—a characteristic at some stage of prostate cancer progression [37]. Recently solved structure of *hc*PheRS [17] demonstrated existences of many noncatalytic function domains, which are involved in a variety of biological functions in other proteins. Insight into the functional role of these unique modules and revealing their relationship to the etiology of cancer, if any, is still to be determined.

## Figures and Tables

**Table 1:** Table of division of aaRSs into classes.

Class I aaRSs	Class II aaRSs
ValRS, LeuRS, IleRS, CysRS,	SerRS, ThrRS, ProRS, GlyRS,
MetRS, ArgRS, GluRS, GlnRS,	HisRS, AspRS, AsnRS, PheRS,
TyrRS, TrpRS, LysRS I	AlaRS, LysRS II
	SepRS, PylRS, *hm*PheRS

Signature motifs	
HIGH, KMSKS	motif 1, motif 2, motif 3

Architecture of catalytic domains	
Rossmann fold	Antiparallel fold

Primary site of aminoacylation	
2′-OH	3′-OH (except of PheRS)

## Abbreviations

aaRS: Aminoacyl-tRNA synthetase;
PheRS: Phenylalanyl-tRNA synthetase (other specific aminoacyl-tRNA synthetases are denoted by their three-letter amino acid designation);
*tt*: *Thermus thermophilus*;
*hc*: Human cytosolic;
*hm*: human mitochondrial;
*ph*: *Pyrococcus horikoshii*;
ROS: Reactive oxygen species;
ABD: Anticodon-binding domain;
CAM: catalytic module;
CLM: Catalytic-like module;
HTH: Helix-turn-helix;
DBD: DNA/RNA binding domain;
*K*_*m*_: Michaelis constant.

## References

[1] Safro MG, Moor NA (2009). Codases: 50 years after. *Molecular Biology*.

[2] Ibba M, Soll D (2000). Aminoacyl-tRNA synthesis. *Annual Review of Biochemistry*.

[3] Eriani G, Delarue M, Poch O, Gangloff J, Moras D (1990). Partition of tRNA synthetases into two classes based on mutually exclusive sets of sequence motifs. *Nature*.

[4] Ling J, Reynolds N, Ibba M (2009). Aminoacyl-tRNA synthesis and translational quality control. *Annual Review of Microbiology*.

[5] Kotik-Kogan O, Moor N, Tworowski D, Safro M (2005). Structural basis for discrimination of L-phenylalanine from L-tyrosine by phenylalanyl-tRNA synthetase. *Structure*.

[6] Fishman R, Ankilova V, Moor N, Safro M (2001). Structure at 2.6 Å resolution of phenylalanyl-tRNA synthetase complexed with phenylalanyl-adenylate in the presence of manganese. *Acta Crystallographica D*.

[7] Mosyak L, Reshetnikova L, Goldgur Y, Delarue M, Safro MG (1995). Structure of phenylalanyl-tRNA synthetase from *Thermus thermophilus*. *Nature Structural Biology*.

[8] Goldgur Y, Mosyak L, Reshetnikova L (1997). The crystal structure of phenylalanyl-tRNA synthetase from *Thermus thermophilus* complexed with cognate tRNA^Phe^. *Structure*.

[9] Moor N, Kotik-Kogan O, Tworowski D, Sukhanova M, Safro M (2006). The crystal structure of the ternary complex of phenylalanyl-tRNA synthetase with tRNA^Phe^ and a phenylalanyl-adenylate analogue reveals a conformational switch of the CCA end. *Biochemistry*.

[10] Khodyreva SN, Moor NA, Ankilova VN, Lavrik OI (1985). Phenylalanyl-tRNA synthetase from *E. coli* MRE-600: analysis of the active site distribution on the enzyme subunits by affinity labelling. *Biochimica et Biophysica Acta*.

[11] Sanni A, Walter P, Boulanger Y, Ebel J-P, Fasiolo F (1991). Evolution of aminoacyl-tRNA synthetase quaternary structure and activity: Saccharomyces cerevisiae mitochondrial phenylalanyl-tRNA synthetase. *Proceedings of the National Academy of Sciences of the United States of America*.

[12] Klipcan L, Levin I, Kessler N, Moor N, Finarov I, Safro M (2008). The tRNA-induced conformational activation of human mitochondrial phenylalanyl-tRNA synthetase. *Structure*.

[13] Yadavalli SS, Klipcan L, Zozulya A (2009). Large-scale movement of functional domains facilitates aminoacylation by human mitochondrial phenylalanyl-tRNA synthetase. *FEBS Letters*.

[14] Rodova M, Ankilova V, Safro MG (1999). Human phenylalanyl-tRNA synthetase: cloning, characterization of the deduced amino acid sequences in terms of the structural domains and coordinately regulated expression of the *α* and *β* subunits in chronic myeloid leukemia cells. *Biochemical and Biophysical Research Communications*.

[15] Moor N, Linshiz G, Safro M (2002). Cloning and expression of human phenylalanyl-tRNA synthetase in *Escherichia coli*: comparative study of purified recombinant enzymes. *Protein Expression and Purification*.

[16] Guo M, Schimmel P, Yang X-L (2009). Functional expansion of human tRNA synthetases achieved by structural inventions. *FEBS Letters*.

[17] Finarov I, Moor N, Kessler N, Klipcan L, Safro MG (2010). Structure of human cytosolic phenylalanyl-tRNA synthetase: evidence for kingdom-specific design of the active sites and tRNA binding patterns. *Structure*.

[18] Kavran JM, Gundllapalli S, O’Donoghue P, Englert M, Söll D, Steitz TA (2007). Structure of pyrrolysyl-tRNA synthetase, an archaeal enzyme for genetic code innovation. *Proceedings of the National Academy of Sciences of the United States of America*.

[19] Roy H, Ling J, Irnov M, Ibba M (2004). Post-transfer editing in vitro and in vivo by the *β* subunit of phenylalanyl-tRNA synthetase. *The EMBO Journal*.

[20] Sasaki HM, Sekine S-I, Sengoku T (2006). Structural and mutational studies of the amino acid-editing domain from archaeal/eukaryal phenylalanyl-tRNA synthetase. *Proceedings of the National Academy of Sciences of the United States of America*.

[21] Lin SX, Baltzinger M, Remy P (1984). Fast kinetic study of yeast phenylalanyl-tRNA synthetase: role of tRNA^Phe^ in the discrimination between tyrosine and phenylalanine. *Biochemistry*.

[22] Roy H, Ling J, Alfonzo J, Ibba M (2005). Loss of editing activity during the evolution of mitochondrial phenylalanyl-tRNA synthetase. *Journal of Biological Chemistry*.

[23] Klipcan L, Moor N, Kessler N, Safro MG (2009). Eukaryotic cytosolic and mitochondrial phenylalanyl-tRNA synthetases catalyze the charging of tRNA with the *meta*-tyrosine. *Proceedings of the National Academy of Sciences of the United States of America*.

[24] Schmitt E, Mechulam Y, Fromant M, Plateau P, Blanquet S (1997). Crystal structure at 1.2 Å resolution and active site mapping of *Escherichia coli* peptidyl-tRNA hydrolase. *The EMBO Journal*.

[25] Goodall JJ, Chen GJ, Page MGP (2004). Essential role of histidine 20 in the catalytic mechanism of *Escherichia coli* peptidyl-tRNA hydrolase. *Biochemistry*.

[26] Wang L, Schultz PG (2004). Expanding the genetic code. *Angewandte Chemie International Edition*.

[27] Dean RT, Fu S, Stocker R, Davies MJ (1997). Biochemistry and pathology of radical-mediated protein oxidation. *Biochemical Journal*.

[28] Fu S, Davies MJ, Stocker R, Dean RT (1998). Evidence for roles of radicals in protein oxidation in advanced human atherosclerotic plaque. *Biochemical Journal*.

[29] Ibba M, Kast P, Hennecke H (1994). Substrate specificity is determined by amino acid binding pocket size in *Escherichia coli* phenylalanyl-tRNA synthetase. *Biochemistry*.

[30] Sharma N, Furter R, Kast P, Tirrell DA (2000). Efficient introduction of aryl bromide functionality into proteins in vivo. *FEBS Letters*.

[31] Kirshenbaum K, Carrico IS, Tirrell DA (2002). Biosynthesis of proteins incorporating a versatile set of phenylalanine analogues. *Chembiochem*.

[32] Datta D, Wang P, Carrico IS, Mayo SL, Tirrell DA (2002). A designed phenylalanyl-tRNA synthetase variant allows efficient in vivo incorporation of aryl ketone functionality into proteins. *Journal of the American Chemical Society*.

[33] Lee JW, Beebe K, Nangle LA (2006). Editing-defective tRNA synthetase causes protein misfolding and neurodegeneration. *Nature*.

[34] Oki K, Sakamoto K, Kobayashi T, Sasaki HM, Yokoyama S (2008). Transplantation of a tyrosine editing domain into a tyrosyl-tRNA synthetase variant enhances its specificity for a tyrosine analog. *Proceedings of the National Academy of Sciences of the United States of America*.

[35] Wang L, Xie J, Schultz PG (2006). Expanding the genetic code. *Annual Review of Biophysics and Biomolecular Structure*.

[36] Sen S, Zhou H, Ripmaster T, Hittelman WN, Schimmel P, White RA (1997). Expression of a gene encoding a tRNA synthetase-like protein is enhanced in tumorigenic human myeloid leukemia cells and is cell cycle stage- and differentiation-dependent. *Proceedings of the National Academy of Sciences of the United States of America*.

[37] Vellaichamy A, Sreekumar A, Strahler JR (2009). Proteomic interrogation of androgen action in prostate cancer cells reveals roles of aminoacyl tRNA synthetases. *PLoS One*.

